# The fate of thoracolumbar surgeries in patients with Parkinson’s disease, and analysis of risk factors for revision surgeries

**DOI:** 10.1186/s12891-019-2481-8

**Published:** 2019-03-14

**Authors:** Huan Sheu, Jen-Chung Liao, Yu-Chih Lin

**Affiliations:** Department of Orthopedics Surgery, Bone and Joint Research Center, Chang Gung Memorial Hospital, Chang Gung University, No._5, Fu-Shin Street, Kweishian, Taoyuan, 333 Taiwan

**Keywords:** Parkinson’s disease, Spinal instrumented surgery, Complications, Revision surgery

## Abstract

**Background:**

Compared to patients without Parkinson’s disease (PD), patients with PD who underwent spinal surgeries were reported to have a relatively high complication rate. However, studies that analyze surgical risk factors for these patients are limited.

**Methods:**

From October 2004 to April 2015, patients with PD who underwent spinal surgeries at our department were reviewed. Patients who underwent lumbar or thoracolumbar instrumented surgeries due to degeneration or deformity disease were included. Any reason for revision surgery was recorded. Risk factors including patients’ factors, surgical factors, and lumbo-pelvic radiographic parameters were analyzed. Patients’ factors included patients’ underlying diseases, body mass index (BMI), osteoporotic status, and PD’s severity using the modified Hoehn and Yahr staging scale. Surgical factors included surgical levels, extending to thoracic spine or not, corrective osteotomy, with anterior approach or not, and interbody device. Radiographic parameters included lumbar lordosis (LL), sacral slope (SS), pelvic tilt (PT), pelvic incidence (PI), coronal Cobb’s angles, and score for spino-pelvic realignment achievement.

**Results:**

A total of 66 patients were enrolled. The mean age at surgery was 69.0 years old. The mean follow-up time was 51.2 months. Twenty-six revision surgeries were required in 19 patients (29%). Risk factors for revision surgery included modified Hoehn and Yahr stage ≥3 (*p* <  0.001), cancer history (*p* = 0.024), osteoporosis (*P* = 0.012) and underwent corrective osteotomy (*p* = 0.035). According to binary logistic regression analysis, the modified Hoehn and Yahr stage ≥3 (*p* <  0.001) was the only independent risk factor. The Kaplan-Meier analysis revealed patients with long instrumentation (surgical levels > 3), T-spine instrumentation, and lower score of spino-pelvic realignment achievement tended to have earlier revision.

**Conclusion:**

For PD patients planning for elective thoracolumbar surgery, aggressive control status of PD before or after surgery is necessary to prevent surgical complications. Longer surgical levels and corrective osteotomy also tended to have earlier revision. A better score in spino-pelvic realignment achievement after surgery could reduce occurrence of revision.

## Background

Parkinson’s disease (PD) emerges a second common neurodegenerative condition after Alzheimer disease, characterized by tremors, bradykinesia, rigidity and postural instability [1]. In addition, patients with PD usually present with poor bone quality, severe muscular dysfunction and abnormal posture. These sequelae might result in spinal pathology and result in spine operations for some patients with scoliosis, kyphosis, osteoporotic fracture, or degenerative spondylosis [2]. Comparing with the general population, it would be expected that spinal surgeries in PD patients would be prone to fail, necessitating revision surgery. This is the dilemma facing the spine surgeon when treating PD patients with spinal disease. A few reports have documented that outcomes for PD patients after spine surgeries are not satisfactory because of a high complication rate. Koller et al. described a series of 23 patients that had a 35% reoperation rate [3]. Bourghli et al. reported 12 PD patients with long fusion, and half the patients eventually underwent revision [4]. Kimura et al. reported a multicenter retrospective study which contained 67 PD patients who underwent lumbar surgery, divided into three groups (laminectomy, fusion, corrective surgery) according to their surgical methods [5]. The results showed the surgical failure rate was higher in the fusion group and in the corrective surgery group than in the laminectomy only group; and a small preoperative lumbar lordosis angle was associated with a failure of the initial surgery. The risk factors for revision spinal surgery in PD patients undergoing instrumented thoracolumbar surgery are not well defined due to the small study series with limited number of patients. Because the data concerning about risk factors for PD patients underwent spine surgery are still limited, the optimal guideline for surgically treating this kind of patient remains inconsistent. In this study, we reviewed the clinical and radiographic results of PD patients who underwent thoracolumbar or lumbar instrumented fusion procedures at our department, and analyzed the risk factors leading to revision surgery. The purpose of the study was to identify factors leading to surgical failure in PD patients who underwent instrumented spine surgery, and to develop surgical strategy for this kind of patients.

## Methods

After obtaining the approval from the institutional review board, we retrospectively reviewed patients with PD who underwent spinal surgeries between October 2004 and April 2015 at our institute. The inclusion criteria was PD patients who were underwent decompression with instrumented thoracolumbar or lumbar arthrodesis. The main pathology for surgery was spinal stenosis with lumbar or thoracolumbar instability, including degenerative lumbar spondylolisthesis, degenerative lumbar scoliosis, degenerative lumbar kypho-scoliosis, degenerative thoracolumbar kypho-scoliosis, and adjacent segment instability after previous surgery. We excluded index surgeries for infection or tumor. In the present study, all patients underwent conventional open surgeries, either posterior approach only or anterior combined with posterior approach.

The demographic data of all the study participants, including age, gender, comorbidities, osteoporotic status (World Health Organization definition, T-score < − 2.5), fused segments, and estimated blood loss were collected from their medical records. The preoperative PD severity of these patients was reviewed using the modified Hoehn and Yahr (H&Y) scale. The modified H&Y scale was as follows for stages 1–5, which described the symptom progression of PD.

1. Stage 0: No signs of disease.
2. Stage 1: Symptoms are very mild; unilateral involvement only.
3. Stage 1.5: Unilateral and axial involvement.
4. Stage 2: Bilateral involvement without impairment of balance.
5. Stage 2.5: Mild bilateral disease with recovery on pull test.
6. Stage 3: Mild to moderate bilateral disease, some postural instability, but physically independent.
7. Stage 4: Severe disability, but still able to walk or stand unassisted.
8. Stage 5: Wheelchair bound or bedridden unless aided.

Preoperative plain radiographs (lateral, anteroposterior, and flexion-extension) and magnetic resonance imaging were used to assess the spondylolisthesis, degenerative coronal or sagittal deformity, and spinal stenosis. Postoperative plain radiographs and magnetic resonance imaging were used to evaluate new fractures, implant-related complications and adjacent stenosis or instability.

All the causes of revision surgeries were recorded during the study. Furthermore, the risk factors were grouped into 3 categories: patient-related factors, surgery-related factors and radiographic parameters. The patient-related factors included comorbidities, such as diabetes mellitus, hypertension, chronic kidney disease, ischemic heart disease and cancer history; osteoporotic status and PD severity (modified H&Y stage) were also analyzed. The surgery-related factors included blood loss, surgical level, thoracic spine involvement, corrective osteotomy, interbody device placement, and surgical approach (posterior approach only or combined posterior and anterior approach). The radiographic parameters including the lumbar lordosis (LL), sacral slope (SS), pelvic tilt (PT), pelvic incidence (PI) and score for spino-pelvic realignment achievement (score 0, 1, 2) were recorded. LL angle was measured using Cobb method of upper endplate of L1-S1; SS angle was measured the angle between the sacral endplate and a horizontal line; PT angle was measured by the line through midpoint of sacral plate and midpoint of femoral heads axis, and the vertical line; PI angle measured by the line through midpoint of sacral line and midpoint of femoral heads axis, and the line vertical to sacral plate [6]. The achievement of a successful harmony of spino-pelvic realignment had been mentioned by Schwab et al. [7]. The ideal realignment objectives in the sagittal plane included sagittal vertical axis (SVA) < 50 mm, PT < 20° and LL = PI ±9°. Based on these objectives, we developed a scoring system to evaluate the spino-pelvic achievement. The total score ranged from 0 to 2. Due to lack of data base of SVA < 50 mm, we only evaluate the achievement of PT < 20° and LL = PI ±9° on every patient. The patient with PT < 20° would get 1 point, if not, get nothing. In the same way, the patient with LL = PI ±9°, would get 1 point, if not, get nothing. Thus, the total score ranges from 0 to 2. Higher scores represent better achievement of spino-pelvic harmony. Table 1 demonstrated the definition of the spino-pelvic realignment achievement score used in this study.

**Table 1:** The spino-pelvic realignment achievement

Score	Condition
0	PT ≥ 20° and LL ≠ PI ±9°
1	PT < 20° or LL = PI ±9°
2	PT < 20° and LL = PI ±9°

*PT* pelvic tilt, *LL* lumbar lordosis, *PI* pelvic index

### Statistical analysis

The Chi-square test and Fisher’s exact test were used for categorical variables. The Student t test was used for continuous variables. A binary logistic regression model was performed to analyze risk factors, including the most independent risk factors. Kaplan-Meier analysis was used to estimate the cumulative incidence of revision. A two-tailed value of *p* < 0.05 was considered statistically significant.

## Results

Initially a total of 88 patients were included in this study. Five patients who lost to follow-up at orthopedic clinics, and the other 17 patients were excluded because of unrecognizable modified H&Y stage by medical records. Therefore, 66 patients (22 males and 44 females) were enrolled into the study. The mean patient age was 69.0 ± 7.2 years old. The mean follow-up period was 51.2 ± 30.5 months. All the patients took dopamine agonist medication rather than deep brain stimulation. The PD severity distribution showed 11 patients with modified H&Y stage 1, 11 patients with stage 1.5, 12 patients with stage 2, 6 patients with stage 2.5, 20 patients with stage 3, and 6 patients with stage 4. The index surgical indications were spinal stenosis with degenerative lumbar spondylolisthesis, degenerative lumbar scoliosis, or thoracolumbar kyphoscoliosis. The total revision rate was 29.0% (19/66), of which 26 revision surgeries were required in these 19 patients. On the other hand, 71% of patients (47/66) were stable and did not need revision surgery during their follow-up period. The demographic data are listed in Table 2. The ratio of patients with a modified H&Y stage ≥3 was greater in the revision group (16/19) than in the non-revision group (10/47; *p* < 0.001). The ratio of the patients in the revision group who had cancer history (2/19 vs. 0/47, *p* = 0.024) or osteoporotic status (10/19 vs. 10/47, *p* = 0.012) were also higher. The gender ratios and respective surgical levels were similar between the revision and non-revision groups. The other comorbidities in the revision group, including diabetes mellitus, hypertension, chronic kidney disease and ischemic heart disease were at identical levels to those in the non-revision group. Blood loss during surgery was comparable in both groups. The revision group had a significantly higher ratio of patients who underwent corrective osteotomy than non-revision group (3/19 vs. 1/47, *p* = 0.035). Though statistically non-significant, the revision group had a higher proportion of patients with surgical levels > 3 (12/19 vs. 19/47, *p* = 0.094), instrumentation extending to the thoracic spine (3/19 vs. 2/47, *p* = 0.019) and who underwent anterior approach surgery (1/19 vs. 2/47, *p* = 0.859). There was a lower prevalence of interbody spine fusion surgery in the revision group (8/19 vs. 29/47, *p* = 0.146). Regarding radiographic parameters, the pre-operative and post-operative LL, SS, PT and PI did not differ between the revision and non-revision groups. However, the revision group consisted of a higher ratio of patients who did not achieve post-operative spino-pelvic harmony. There were only 21.1% with LL = PI ±9°, 26.3% with PT < 20° and 10.5% with a satisfactory spino-pelvic realignment achievement score, compared with those in the non-revision group whose patients achieve 42.6, 42.6 and 27.7%, respectively.

**Table 2:** Patient demographic data (Non-revision group vs. revision group)

	Non-revision group (*n* = 47)	Revision group (*n* = 19)	*P* value
Patient-related factors			
Age	70.43 ± 7.39	66.74 ± 6.28	0.060
Gender (M/F)	17/30	5/14	0.442
DM	11 (23.4)	5 (26.3)	0.803
HTN	29 (61.7)	10 (52.6)	0.497
CKD	0 (0)	1 (5.3)	0.113
IHD	7 (14.9)	1 (5.3)	0.278
Cancer	0 (0)	2 (10.5)	0.024*
Osteoporosis	10 (21.3)	10 (52.6)	0.012*
Modified H&Y stage			< 0.001*
1	11	0	
1.5	10	1	
2	11	1	
2.5	5	1	
3	8	12	
4	2	4	
Surgery-related factors			
Blood loss (cc)	1072.55 ± 1094.20	989.47 ± 836.26	0.767
Surgical levels			0.094
≤ 3	28 (59.6)	7 (36.8)	
> 3	19 (40.4)	12 (63.2)	
T-spine instrumentation	2 (4.3)	3 (15.8)	0.109
Interbody fusion	29 (61.7)	8 (42.1)	0.146
Corrective osteotomy	1 (2.1)	3 (15.8)	0.035*
Combined anterior approach	2 (4.3)	1 (5.3)	0.859
Radiographic parameters			
Pre OP-			
LL	37.20 ± 17.29	42.75 ± 14.15	0.220
SS	29.39 ± 9.44	32.67 ± 5.92	0.165
PT	22.09 ± 10.40	22.81 ± 9.31	0.794
PI	51.67 ± 10.40	55.48 ± 10.08	0.179
Post OP			
LL	36.39 ± 14.08	38.74 ± 10.23	0.512
SS	29.89 ± 9.21	30.17 ± 6.61	0.905
PT	21.31 ± 9.13	21.81 ± 8.21	0.836
PI	51.2 0 ± 9.31	51.98 ± 8.48	0.753
LL = PI ±9	20 (42.6)	4 (21.1)	0.100
PT < 20	20 (42.6)	5 (26.3)	0.218
Spino-pelvic realignment achievement			
0	20 (42.6)	12 (63.2)	0.224
1	14 (29.8)	5 (26.3)	
2	13 (27.7)	2 (10.5)	

*DM* Diabetes mellitus, *HTN* Hypertension, *CKD* Chronic kidney disease, *IHD* Ischemic heart disease, *modified H&Y stage* modified Hoehn and Yahr stage, *T-spine* Thoracic spine, *LL* Lumbar lordosis, *SS* Sacral slope, *PT* Pelvic tilt, *PI* Pelvic incidence

The reasons for revision were listed as Table 3. Eight patients suffered from implant loosening or loss of screws, 7 patients developed an instrumented vertebral fracture, another 7 patients developed new compression fractures adjacent to the upper or lower instrumented vertebra, 3 patients showed adjacent stenosis or instability, and 1 patient had a wound infection.

**Table 3:** The reasons for revision surgery

	Number (%)
Etiology	(26 operations in 19 cases)
Hardware failure	8 (30.8)
Instrumented fracture	7 (26.9)
Compression fracture	7 (26.9)
Adjacent stenosis or instability	3 (11.5)
Infection	1 (3.8)

Based on the demographic data mentioned above, the risk factors that cause revision episodes were analyzed. For patient-related factors, the patients with a modified H&Y stage ≥3 (OR = 19.73, *p* < 0.001), cancer history (*p* = 0.024) or osteoporotic status (OR = 4.11, *p* = 0.012) had a higher incidence of revisions. For surgery-related factors, the patients who underwent corrective osteotomy had a significantly higher revision rate (OR = 8.63, *p* = 0.035). There was no significant difference between the groups for lumbo-pelvic radiographic parameters. These significant risk factors were put into a logistic regression model and survival analysis to determine which was the most independent. In binary logistic regression analysis, a modified H&Y stage ≥3 (expected value = 0.05, confidence interval 0.11~0.24, *p* < 0.001) was the only independent risk factor (Table 4).

**Table 4:** The binary logistic regression model

	Exp. (CI)	P value
Modified H&Y stage ≥3	0.05 (0.11~0.24)	< 0.001*
Cancer	0.000	0.999
Osteoporosis	0.27 (0.53~1.36)	0.112
Corrective osteotomy	0.51 (0.20~13.82)	0.688

*Exp*. Exponential function, *CI* Confidence interval

**Fig. 1:**
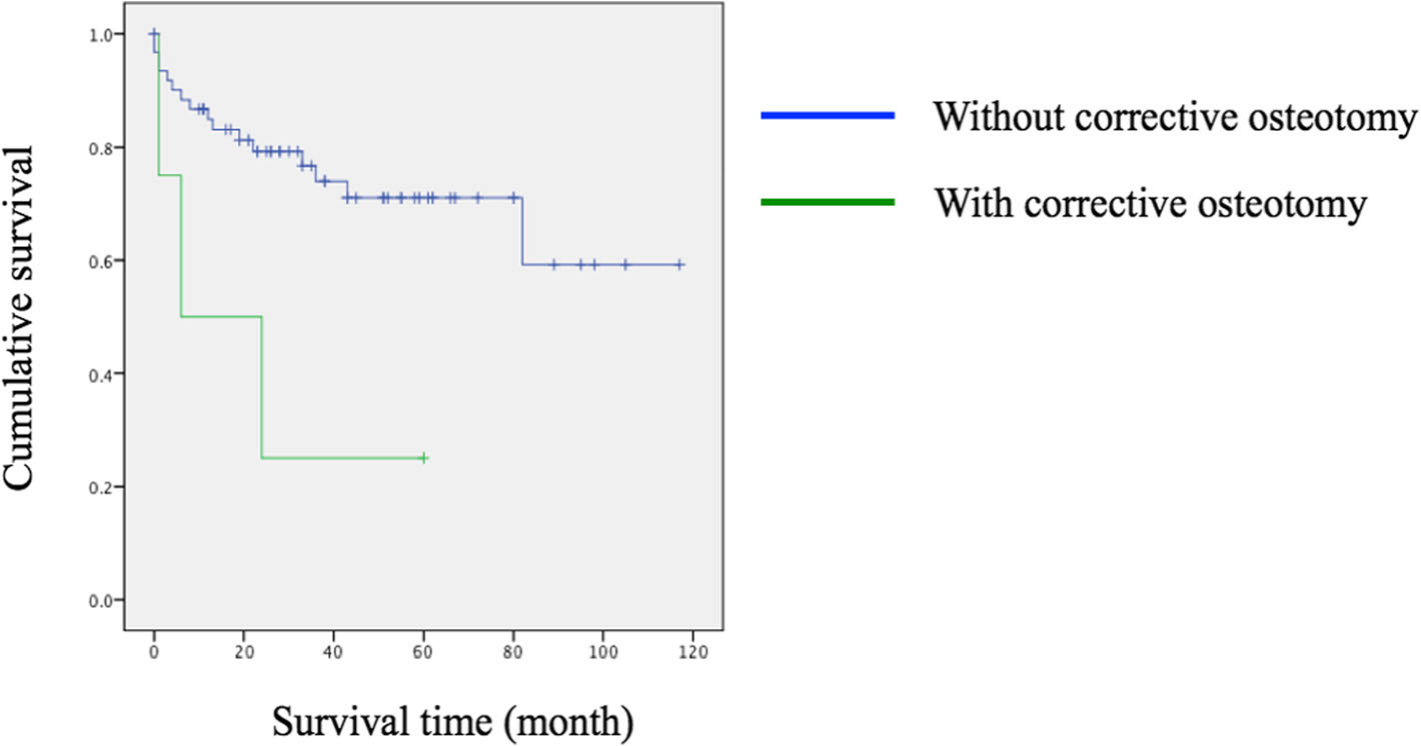
Kaplan-Meier curves for patients stratified by receiving osteotomy or not. Green line represented those with osteotomy; blue line represented those without osteotomy, *p* = 0.020

**Fig. 2:**
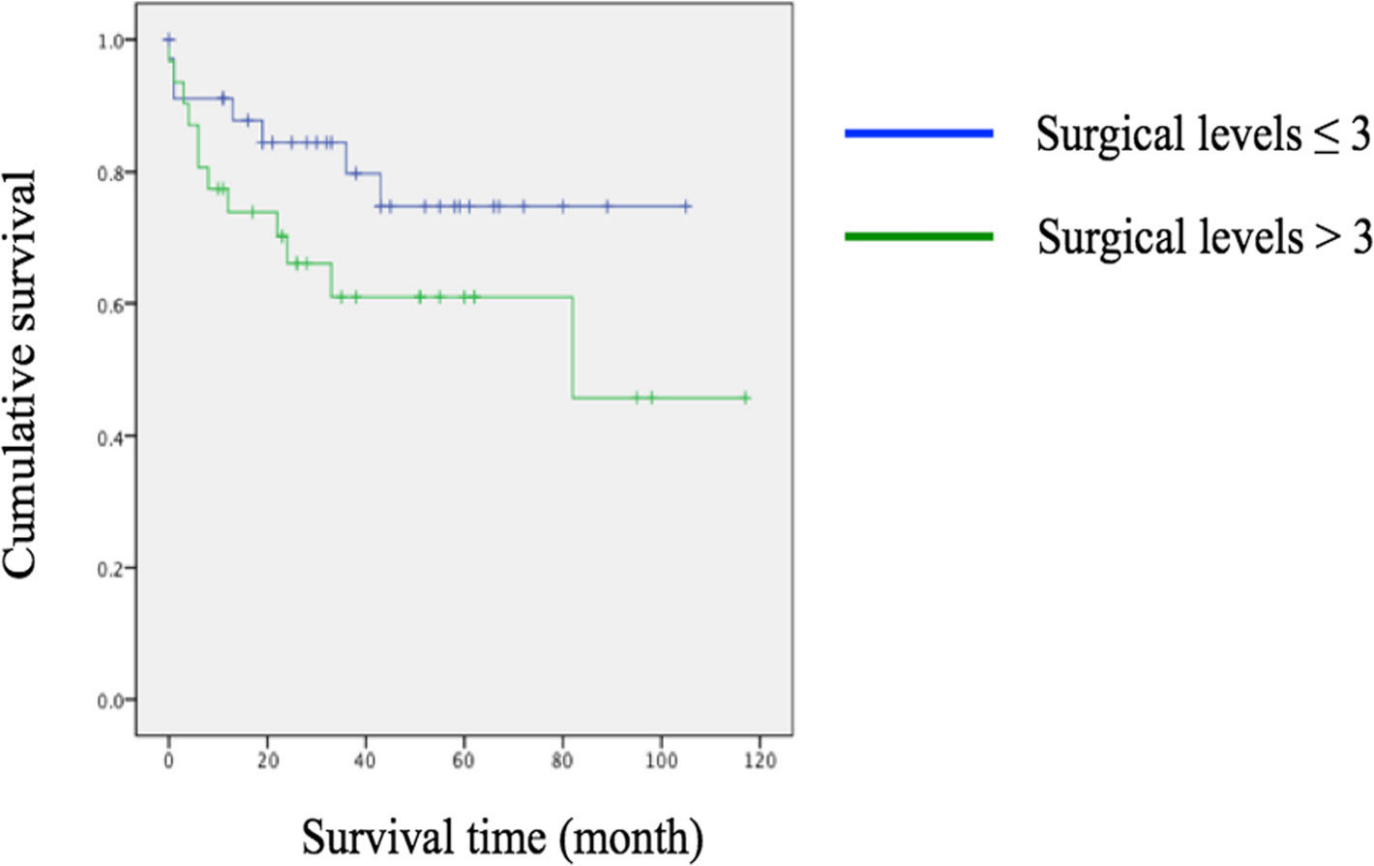
Kaplan-Meier curves for patients stratified by surgical levels. Green line represented over three levels; Blue line represented less or equal to three levels, *p* = 0.136

**Fig. 3:**
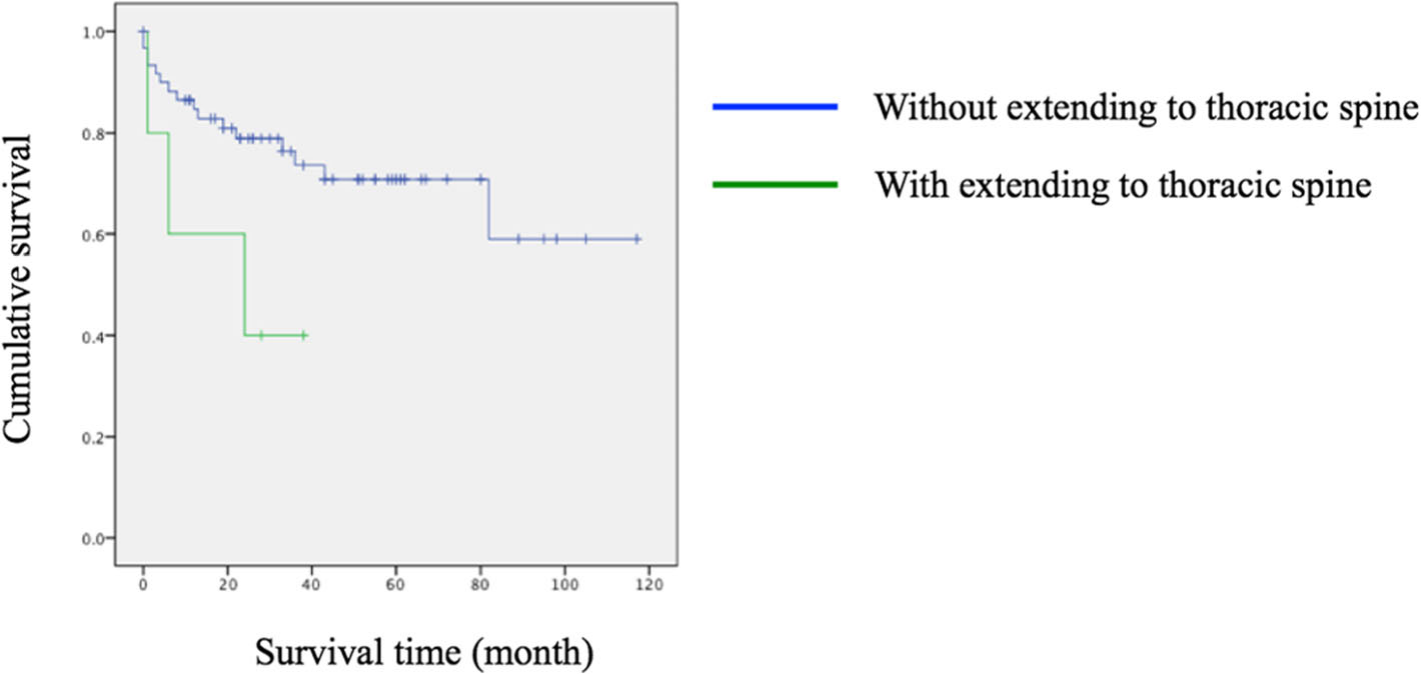
Kaplan-Meier curves for patients stratified by instrumentation to thoracic vertebrae or not. Green line represented those with instrumentation to thoracic spine; blue line represented those without instrumentation to thoracic spine, *p* = 0.065

**Fig. 4:**
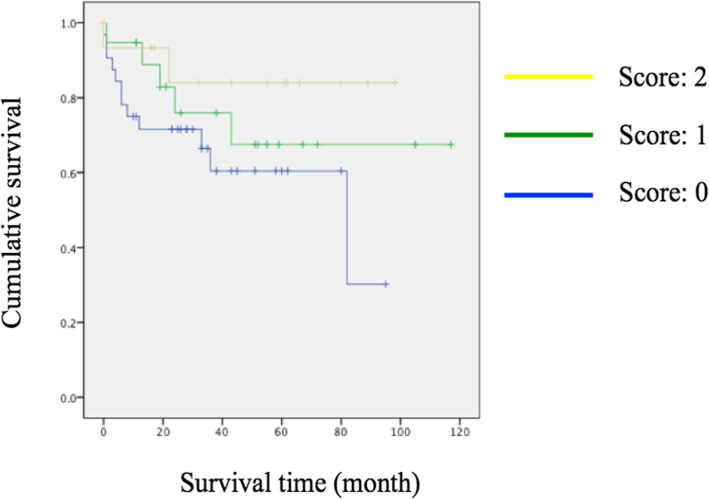
Kaplan-Meier curves for patients stratified by spino-pelvic realignment achievement score. Blue line = score 0; green line = score 1; yellow line = 2, *p* = 0.241

Kaplan-Meier analysis revealed significant early revision surgeries were inevitable in those with corrective osteotomy (*p* = 0.020) (Fig. 1). In spite of no significance, there was also a trend for earlier revision in those with long instrumentation (surgical levels > 3; *p* = 0.136) (Fig. 2), surgery extending to the thoracic spine (*p* = 0.065) (Fig. 3), and a lower score on the spino-pelvic realignment achievement (*p* = 0.241) (Fig. 4).

## Discussion

Recent meta-analysis have shown a rising prevalence of PD with age (all per 100,000): 428 for ages 60–69 years; 425 for ages 65–74 years; 1087 for ages 70–79 years; and 1903 in those older than age 80 [8]. In addition, advancement in medical care and public health has led to a rapidly growing geriatric population, those of which continue to lead active lives well into their eighth and ninth decade. As such, the number of PD patients with complicated spine problems who need to be treated will increase.

PD patients present with decreased bone quality and postural dysfunction. A meta-analysis conducted by Zhao et al. concluded that PD patients were at higher risk of osteoporosis than healthy controls (OR 1.18, 95% CI: 1.09–1.27) [9]. Another meta-analysis measuring the same parameters found similar results (OR 2.61, 95% CI: 1.69–4.03) [10]. In addition, other studies suggest that the relationship between PD and low bone density is associated with the H&Y stage, and to the duration of disease evolution [11–14]. Several mechanisms may contribute to PD-related bone loss, including weight loss, immobilization, Vitamin D levels, L-dopa therapy and dietary deficiency [15]. In our study, the revision group was composed of a significantly higher ratio of patients with osteoporosis and those with severe modified H&Y stage scores. Indeed, modified H&Y stage was the only independent risk factor for revision observed in our study, which suggests these two risk factors were positively associated or confounding.

Sagittal deformities of PD including camptocormia and antecollis, can be contributed to by muscular rigidity, axial dystonia, weakness caused by myopathy, body scheme defects due to centrally impaired proprioception, and by structural changes in the spine [16]. Oh et al. found 42% of PD patients had significant sagittal mal-alignment using the threshold of SVA > 50 mm, and 51% of those with PD had spino-pelvic mismatch (PI-LL > 10°) [17]. Bissolotti et al. analyzed 31 consecutive PD patients in a cross-sectional study, focusing on sagittal alignment [18]. Although the anatomical parameter PI was similar, the functional PT appeared to be increased and SS decreased when compared to the healthy adult cohort. In another retrospective study of 175 PD cases, they found male gender, longer disease duration, higher H&Y class, and a low plumb line-L3 distance were negative factors for spinal imbalance and risk of falling [19]. In our study, patients with PD in both the revision and non-revision group had abnormal lumbo-pelvic radiographic parameters compared with the general population (decreased LL, increased PT and decreased SS), although this could be related to age and degenerative change as well. Given these reports, spinal surgery would be a more difficult task in PD patients, than in those without PD.

The previous studies reported the revision rates in PD patients who had undergone spinal surgeries were from 21 to 86% [2–4, 7]. In our study, the revision rate was 29%, and the most common reasons for revision surgeries included hardware failure, instrumented fracture and compression fracture. All of these could be related to poor bone quality or progressive postural abnormality, a similar conclusion drawn by Babat et al., who reported that of 14 patients that underwent spine surgery, 12 (86%) required additional surgery, undergoing a total of 31 reoperations [20].

To improve postoperative satisfaction, some authors suggested that restoration of spinopelvic balance is paramount. Koller et al. described a series of 23 PD patients, in which 78% were satisfied or very satisfied despite a high rate of reoperations (35%); and proposed that the reconstruction of physiological lumbar lordosis and lumo-pelvic parameters was the key to prevent failure of surgery [3]. Postoperative or follow-up sagittal imbalance (C7-sagittal center vertical line; C7-SVL > 10 cm) had a significantly increased rate of revision surgery (*p* = 0.031). Bourghli et al. also echo this concept [4]. They concluded that long posterior instrumentation and fusion, from T2 to the pelvis, can restore sagittal and frontal imbalance, providing good clinical and radiographic results over the intermediate term with a high rate of satisfaction, despite 17% proximal junctional kyphosis rate and 50% revision rate.

So far, no ideal radiographic objectives allow surgeons to follow a PD patient’s course peri-operatively. We incorporated the concept of Schwab et al. to establish a scoring system, thus enable us to evaluate peri-operative spino-pelvic realignment achievement. Schwab et al. concluded that the following parameters during surgical intervention can achieve successful patient-specific spinopelvic realignment in the sagittal plane [7]. First of all, global spinal realignment should attempt to achieve a postoperative SVA < 50 mm, to attenuate the feeling of “falling forward.” Second, a PT < 20° is required during efficient ambulation. Finally, LL = PI ±9° may be used to achieve patient-specific alignment. It is evident that the goal of ideal spino-pelvic alignment cannot be obtained in all cases because of a number of limitations. We analyzed not only the spino-pelvic parameters peri-operatively, but also the objective score of spino-pelvic realignment achievement (score 0, 1, 2), to assess whether the realignment was necessary. Because this is a retrospective study and the SVA was not obtained for all patients, we did not use SVA as part of scoring system variables. There was no significant difference in preoperative or postoperative lumbo-pelvic radiographic parameters and score for spino-pelvic realignment achievement. Although Kaplan-Meier analysis showed no significance, we did notice a trend that lower scores led to earlier revisions. These findings suggest surgeons should maintain spino-pelvic harmony as much as possible.

Appropriate selection of surgical indications and awareness of possible risk factors may improve the outcomes of spinal surgeries. Increasing numbers of recent reports noticed the importance of the nature of PD itself on surgical outcomes. Moon et al. reported 20 patients with short lumbar fusions with poor surgical outcomes due to the progressive nature of PD [2]. Therefore, medical or surgical management of PD itself was also important to improve the outcomes of spinal surgery. Schroeder et al. reported 96 patients underwent lumbar spine surgery, but only 63 patients underwent instrumented surgeries [21]; the risk factors for additional surgery (*p* < 0.05) included an H&Y stage ≥3, a history of diabetes mellitus, treatment for osteoporosis, a combined anterior and posterior approach, the use of bone morphogenetic proteins (BMP), and the use of a spine interbody device. In a series of 48 PD patients with spinal deformity described by Bouyer et al., the rate of surgical revision was 42% and a well-balanced pre-operative condition was the only factor associated with optimal results [22]. In our study, the risk factors for revision surgery included modified H&Y stage ≥3, osteoporosis and a corrective osteotomy during surgery. In the binary logistic regression analysis, the modified H&Y stage ≥3 (*p* < 0.001) was the only independent risk factor. In other words, aggressive control of PD before and after surgery is necessary to prevent surgical complications. The Kaplan-Meier analysis revealed a trend for earlier revision in those with extensive surgical correction, such as corrective osteotomy, long instrumentation (surgical levels > 3), surgery extending to the thoracic spine, and a lower score of spino-pelvic realignment achievement.

There were a number of limitations in this study. The main limitation of this retrospective study was the limited number of participants, although the current study had the largest number of PD patients who underwent instrumented thoracolumbar surgery at a single institute to date. Second, variables including SVA and PD medication history could not be completely obtained due to the retrospective nature of the study. Third, the present study was a retrospective review of a heterogeneous patient population with different levels of spine disease, which might have bias and confound results.

## Conclusions

A modified H&Y stage score ≥ 3 was the only independent risk factor leading to revision surgery. For a PD patient planning for elective thoracolumbar surgery, aggressive control of PD before and after surgery is necessary to prevent surgical complications. Corrective osteotomy or longer instrumentation also led to earlier revision. An optimal spino-pelvic realignment after surgery could reduce the occurrence of revision.

## Abbreviations

H&Y scale: Hoehn and Yahr scale
LL: lumbar lordosis
PD: Parkinson’s disease;
PI: pelvic incidence
PT: pelvic tilting
SS: sacral slope
